# Usability and Functional Outcomes of the Obi3 Robotic Feeding Device Among People With Disabilities, Caregivers, and Providers: Mixed Methods Study

**DOI:** 10.2196/86338

**Published:** 2026-08-12

**Authors:** Andrea D Fairman, Ryan B Osal, Heather Keeton, Jonathan P Dekar

**Affiliations:** 1 DESĪN, LLC Jacksonville, FL United States; 2 The Driving Doctor: Adaptive Driving and Rehabilitation Solutions, PC Glendale, RI United States; 3 University of Pittsburgh Pittsburgh, PA United States

**Keywords:** activities of daily living, assistive technology, caregiver, mixed methods study, rehabilitation, robotics, self-feeding, upper extremity, usability study

## Abstract

**Background:**

Loss of self-feeding ability due to severe bilateral upper extremity motor impairment (BUEMI) limits functional independence with a critical activity of daily living, increases medical risks, reduces social participation, and affects overall well-being. Robotic feeding devices are an emerging category of assistive technology. Obi3 is a third-generation, robotic feeding device, regulated in the United States as a Class I medical device and CE marked as a Class I device under the European Union Medical Device Regulation. The device is designed to enable people with BUEMI to independently select and safely consume food and liquid using accessible switches or interfaces while maintaining culturally normative dining practices. Although earlier Obi generations showed promise, comprehensive usability data across multiple stakeholder groups remains limited.

**Objective:**

This study evaluated the usability, safety, and clinical relevance of Obi3 in real-world use from the perspectives of people with BUEMI, caregivers, and rehabilitation providers. The findings were intended to generate evidence to inform clinical decision-making and determinations of medical necessity for potential DME coverage, consistent with the device’s intended use and clinician-assessed implementation.

**Methods:**

A mixed methods usability study provided a 1-week home trial of Obi3. Quantitative measures included the System Usability Scale (SUS), survey items adapted from the Matching Person and Technology (MPT) framework, and self-feeding ratings based on the *International Classification of Functioning, Disability, and Health* impairment scale. Paired-sample *t* tests, Wilcoxon signed-rank tests, and effect sizes (Cohen *d*) assessed changes in self-feeding scores. Qualitative data from open-ended survey responses and follow-up semistructured interviews were analyzed thematically to complement quantitative findings.

**Results:**

A total of 50 participants were enrolled, of whom 42 completed follow-up surveys. Participants included 15 people with BUEMI (pediatric and adult), 14 caregivers, and 13 providers. Participants’ mean self-feeding impairment scores decreased significantly from 3.80 (SD 0.41) at baseline to 0.60 (SD 0.74) post trial (n=15; *t*_14_=10.75; *P*<.001; Cohen *d*=3.40). All patient participants demonstrated improvement in functional self-feeding ability. Mean SUS scores exceeded the benchmark for acceptable usability (≥68) across stakeholder groups: 82.8 (SD 17.2) for people with BUEMI, 85.2 (SD 12.6) for caregivers, and 85.0 (SD 10.7) for providers. Responses to MPT items indicated strong alignment between user goals and device capabilities, perceived safety, and low complexity. Caregivers reported reduced feeding-related workload and stress, and providers endorsed ease of clinical integration. No adverse events or device malfunctions occurred.

**Conclusions:**

Obi3 demonstrated high usability, safety, and clinical use in restoring self-feeding independence among individuals with severe BUEMI. Findings across people with BUEMI, caregivers, and providers support Obi3 as an effective DME that may enhance user autonomy and reduce caregiver burden. Lengthier longitudinal studies are needed to establish long-term clinical efficacy and health outcomes.

## Introduction

### Background

Self-feeding is a fundamental activity of daily living that supports autonomy, nutritional health, and social participation. Within the *International Classification of Functioning, Disability, and Health* (*ICF*) and the *Occupational Therapy Practice Framework*, self-feeding is recognized as a core activity linked to independence, dignity, and quality of life [1,2]. For clarity, this manuscript uses the term self-feeding to refer specifically to bringing food to the mouth, distinguishing it from eating and swallowing*,* as defined by the American Occupational Therapy Association [3]. Loss of self-feeding ability due to bilateral upper extremity motor impairment (BUEMI) affects more than nutritional intake, contributing to reduced autonomy, dignity, and social participation [4,5]. BUEMI arises from conditions such as cerebral palsy, amyotrophic lateral sclerosis, muscular dystrophy, spinal cord injury, and traumatic brain injury, all of which may disrupt voluntary arm and hand function [6-11]. Although caregiver-assisted feeding can maintain nutrition, it does not restore independence and may introduce variability in feeding pace, technique, and safety [12-15]. Over time, reliance on caregiver assistance can increase caregiver burden and reduce quality of life for both individuals and their caregivers [16]. In addition to physical limitations, loss of self-feeding reduces participation in the social and cultural aspects of eating. Mealtimes are important social activities, and dependence on others may limit engagement and contribute to a reduced sense of identity and belonging. Assistive and robotic feeding technologies aim not only to restore physical function but also to support social participation and emotional well-being [17-21].

Powered feeding systems are an emerging class of assistive technology (AT) designed to restore self-feeding ability in individuals with BUEMI [22,23]. In the United States, these devices are regulated as Class I medical devices under US Food and Drug Administration (FDA), as a daily activity assist device, indicating low risk when used as intended [24]. Several powered feeding devices are commercially available, including the Mealtime Partner [25] and Neater Eater systems [26]. Earlier devices typically rely on limited motor control and produce less fluid movement. In contrast, Obi incorporates a 6–degree-of-freedom (DOF) robotic arm designed to enable smoother, more adaptive utensil motion and individualized positioning, reflecting advances in human-centered assistive robotics [27].

Evidence from Al-Halimi and Moussa [28] supports the functional validity of such multi-DOF robotic approaches, demonstrating that individuals with severe BUEMI were able to perform complex daily tasks, including self-feeding and drinking, using a 6-DOF robotic arm mounted on a wheelchair. Their study highlighted individualized control strategies and adaptive movement patterns, underscoring how increased robotic dexterity can restore meaningful interaction and autonomy while self-feeding.

Obi is designed for repeated use, serves a medical purpose, and is appropriate for home use. These characteristics are consistent with the Centers for Medicare & Medicaid Services (CMS) definition of durable medical equipment (DME). Its design for home use further supports its use in long-term care settings. Obi has been widely used in clinical and home settings, with no reported adverse events in major regulatory databases. It has been prescribed by clinicians and reimbursed through multiple funding sources, supporting its role as a clinically recognized intervention for self-feeding impairment [31]. Despite these advances, limited research has evaluated the real-world usability of newer robotic feeding systems across multiple stakeholders. Usability is a key determinant of adoption and sustained use, particularly for devices intended for long-term home use.

Obi has been prescribed by physicians, deemed medically necessary by occupational therapists, and reimbursed by multiple sources, including the Veterans Health Administration, state Medicaid programs, private insurers, and international health systems. These patterns of prescription and funding highlight Obi’s recognition as a legitimate medical intervention to restore self-feeding independence. A recent descriptive study reported that 19 individuals with BUEMI, ranging in age from 8 to 60 years, successfully used an earlier version of Obi in homes, schools, and clinics, achieving independence in food delivery with positive impacts on nutritional intake, autonomy, and social participation [33]. Obi’s third-generation model (Obi3) incorporates user-informed enhancements, including new and expanded utensil options, improved utensil-arm retention, new software features (“reward mode” and “patient power-on”), a 2-inch increase in reach, and faster speeds from scooping to mouth delivery.

The literature and regulatory evidence highlight a critical clinical and societal need for safe, effective, and user-centered technologies that restore self-feeding independence among individuals with BUEMI. The systematic evaluation of Obi3’s usability over a 1-week period, including the enhanced features and perspectives of multiple stakeholders, including participants with BUEMI, caregivers, and providers, has not been conducted previously. Addressing this gap is essential because usability is a key determinant of the adoption and sustained use of assistive technologies in real-world contexts. Usability, defined as the effectiveness, efficiency, and satisfaction with which users achieve goals in specific environments [33], remains particularly critical for devices classified as DME given their intended long-term use.

### Study Purpose and Hypothesis

Building on prior descriptive findings [32], this study conducted a mixed methods usability evaluation of the Obi3 robotic feeding device to assess its performance, safety, and clinical relevance across multiple stakeholder groups in real-world settings. We hypothesized that Obi3 would demonstrate high usability, safety, and clinical use, reflected by System Usability Scale (SUS) scores exceeding the industry benchmark of 68 [34,35], significant improvements in self-feeding impairment scores, and positive qualitative feedback related to satisfaction and integration into daily routines. This approach aimed to generate both quantitative and qualitative evidence to inform iterative device refinement, guide clinical implementation, and support broader adoption through real-world clinical evidence.

## Methods

### Study Design and Oversight

This mixed methods usability study evaluated the Obi3 Robotic Feeding Device across 3 stakeholder groups: people with BUEMI, caregivers, and rehabilitation providers. Quantitative and qualitative methods were combined to determine whether Obi3 fulfills its intended purpose of restoring self-feeding ability for individuals with severe upper-extremity motor impairments and whether it meets formally defined user needs. The study was designed in accordance with the International Organization for Standardization (ISO) 14155:2020 Clinical Investigation of Medical Devices for Human Subjects: Good Clinical Practice and was approved by the WCG Clinical Institutional Review Board (Protocol #20251472).

A target sample of up to 60 participants (20 people with BUEMI, 20 caregivers, and 20 providers) was based on the FDA guidance recommending ≥15 participants per user group for usability or design-validation testing of medical devices [36,37]. The study was coordinated by the device manufacturer, DESĪN LLC, as a single-sponsor remote investigation. All consent procedures, survey administration, and survey data collection were conducted electronically using the HIPAA (Health Insurance Portability and Accountability Act)–compliant Qualtrics platform [38].

### Recruitment and Enrollment

Recruitment followed a structured, multistage process to ensure broad representation while maintaining ethical compliance. Recruitment materials, including flyers and digital notices, were distributed nationally. A triadic recruitment model (patient-caregiver-provider) was prioritized but not required. Providers were eligible if they held professional credentials (eg, occupational therapist, speech-language pathologist, or AT professional [ATP]) and had experience recommending or evaluating assistive technologies for individuals with self-feeding limitations. Caregivers were qualified if they directly assisted an individual with mealtime activities. People with BUEMI were eligible if aged ≥5 years, required assistive feeding technology to increase self-feeding independence, and could provide consent or assent.

Exclusion criteria included residency in California for new Obi users (due to state-mandated 30-day device-trial requirements), inability to provide informed consent, cognitive impairments preventing meaningful participation, or non-English fluency. Certified ATPs employed by DESĪN LLC prescreened potential participants using standard operating procedures aligned with routine clinical practice.

Informed consent (and assent when applicable) was obtained electronically via Qualtrics. Adults, caregivers, and providers electronically signed consent forms; pediatric participants (aged <17 years) provided assent with parental or guardian consent. Verbal consent was documented when physical signing was not feasible. Participants were informed of study procedures, potential risks, and withdrawal rights.

### Obi3 Trial Procedures

Following consent and baseline screening, providers completed the Obi Medical Device Needs Assessment (MDNA) form [38]. The MDNA captured demographics, diagnosis, and self-feeding impairment at baseline and follow-up using the Self-Feeding Impairment Scale. This scale was not intended to serve as a standardized outcome measure and has not undergone formal psychometric validation. Rather, it is a study-specific clinical rating scale embedded within the Obi MDNA form and is conceptually informed by the *ICF* framework. Specifically, the scale reflects levels of functional self-feeding performance ranging from complete dependence to independent performance and was developed to pragmatically characterize the degree of self-feeding impairment observed during routine clinical assessment and device trials. Because the scale was developed internally for clinical use, there are no published studies on its validity, reliability, or responsiveness.

**Figure 1:**
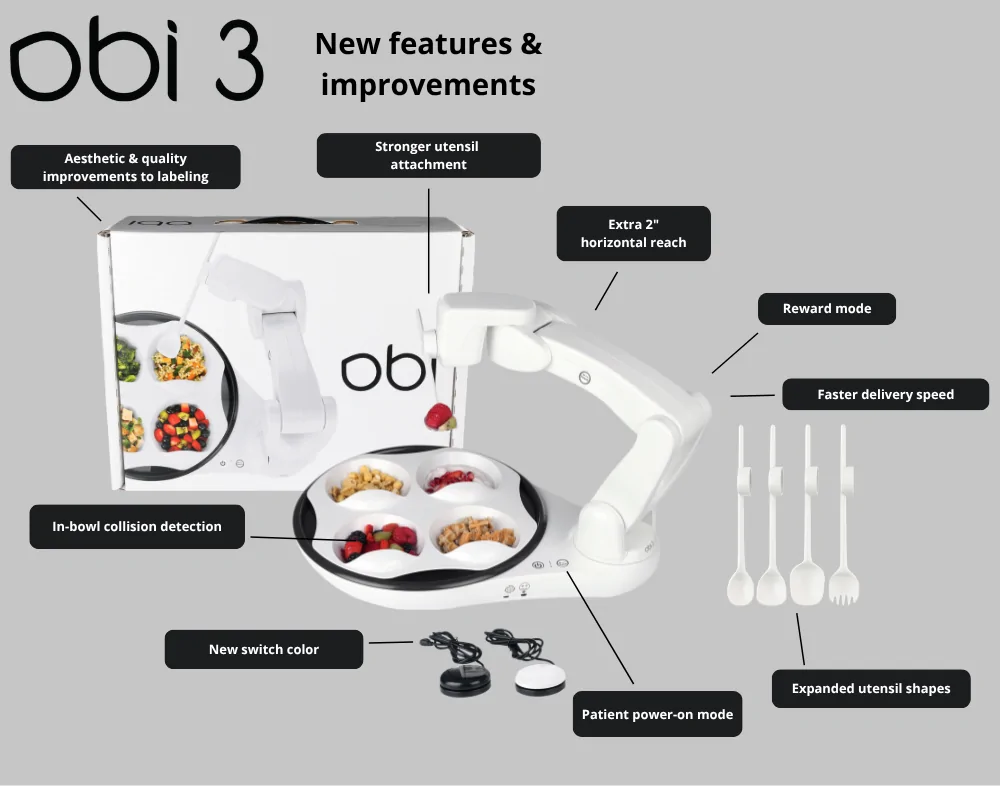
Obi3 robotic feeding device and new features. The Obi3 robotic feeding system includes several user-informed enhancements, such as expanded utensil options, stronger utensil attachment, additional 2-inch horizontal reach, faster delivery speed, reward mode, patient power-on mode, in-bowl collision detection, new switch color, and improved labeling and aesthetic design.

People with BUEMI and their caregivers received an Obi3 unit for a 1-week home trial. The Obi3 (third generation) device incorporated user-informed hardware and software enhancements, expanded utensil options, increased reach, improved utensil-arm retention, and new features such as reward mode and patient power-on (Figure 1).

The trial was conducted in participants’ customary real-world environments, including private homes, outpatient clinics, schools, rehabilitation facilities, and other community-based settings where meals would ordinarily occur, to evaluate how effectively the Obi3 robotic feeding device could be integrated into routine daily activities rather than under controlled laboratory conditions. This approach emphasized ecological validity, allowing researchers to capture authentic user experiences, environmental influences, and contextual factors that affect real-world usability. Each caregiver and patient used the device for a minimum of 5 meals within a 1-week period, providing repeated exposure sufficient to assess learning effects, ease of setup, and consistency of performance across multiple sessions. Providers introduced the Obi3 system using the manufacturer’s *Quick Start Guide* [39], ensuring standardized orientation while permitting flexibility for individualized instruction. Remote technical and clinical support was available throughout the trial to mirror the level of assistance users might receive during early adoption in home or community settings.

Participants interacted with the device in its standard configuration, which included power controls, utensil selection options, and the reward mode feature designed to enhance motivation and engagement. Meals were self-selected to align with each participant’s typical diet, cultural food preferences, and mealtime routines. This design allowed the study to capture performance across a diverse range of food consistencies, environmental contexts, and social settings, thereby providing a more comprehensive understanding of the device’s usability, adaptability, and potential for integration into everyday life.

### Outcome Measures

Electronic surveys were administered to people with BUEMI, caregivers, and providers, with developmentally appropriate adaptations for pediatric participants. Surveys combined standardized and nonstandardized items assessing usability, satisfaction, and integration of Obi3 into daily routines. The primary outcome was the SUS, a 10-item instrument scored 0-100, with 68 as the benchmark for average usability [34,35]. The SUS was administered to adult patients, caregivers, and providers; pediatric participants were excluded because the SUS has not been validated for children. Secondary measures included items adapted from the Matching Person and Technology (MPT) framework, which evaluates personal predisposition, psychosocial fit, and environmental support [40,41]. Adults and teen people with BUEMI (aged ≥15 years) completed the SUS and MPT-based items verifying ≥5 meal exposures and answered nonstandardized questions about instruction clarity, ease of operation, utensil satisfaction, and perceived independence and safety. People with BUEMI aged 15 years and older were grouped within the adult patient category because the survey instruments used in the study included self-report items requiring a higher level of reading comprehension, abstract reasoning, and self-reflection regarding device usability, satisfaction, and independence. These constructs align more closely with adolescent and adult cognitive and communicative abilities than with those of younger children. Grouping patient participants aged 15 years and older with adults allowed for consistent interpretation of survey responses and ensured that the usability and satisfaction data reflected meaningful self-reports.

Younger people with BUEMI (aged 5-14 years) were analyzed separately, as their perspectives were often supplemented or interpreted through caregiver feedback due to differences in literacy, attention span, and cognitive maturity. This stratification thus enhanced the validity and comparability of responses across age groups while maintaining clinical and developmental appropriateness. Pediatric participants with BUEMI (aged <15 years) used simplified, emoji-based Likert items evaluating confidence, enjoyment, and safety.

Caregivers completed survey questions designed to capture both their experience using the Obi device and its impact on daily caregiving demands. These included the SUS [34] to assess perceived ease of use and overall satisfaction; the Zarit Caregiver Burden Scale [42] to evaluate potential changes in emotional, physical, and time-related strain; and a series of custom items addressing device setup, cleaning procedures, and perceived reductions in hands-on feeding time. Together, these instruments provided a comprehensive view of how Obi influenced caregiver workload, stress, and independence for the individual being served.

Providers completed a separate set of survey items, including measures capturing professional background characteristics and impressions of Obi3. They rated the device’s usability and clinical use, including the clarity and completeness of its instructional materials, setup guides, and documentation tools. These data were intended to evaluate the device’s practicality for integration into routine clinical assessment and intervention processes, as well as its suitability for recommendation and long-term use across diverse care settings.

Participants who completed the survey received a US $25 electronic gift card as approved compensation. Optional semistructured interviews were offered to participants following survey completion, whose responses required clarification or elaboration.

### Qualitative Interviews

To complement quantitative data, semistructured virtual interviews were conducted by a researcher independent of DESĪN LLC. Interviews captured lived experiences and stakeholder perspectives, addressing the risk that excluding user voices can yield ineffective technologies. A total of 12 participants were contacted via email, phone, and/or text to elaborate on survey themes. Initial contact with participants who completed the follow-up interviews began the week following survey completion, with recruitment and reminders occurring over a 3-week period. Follow-up interviews were conducted during a limited 4-week period. Early sessions included both provider and pediatric user perspectives, followed by adult user and caregiver interviews later within the 1-month data collection window to ensure accurate recall of the Obi3 trial. Sessions were conducted via Google Meet, with verbal consent to record and transcribe. Transcripts were anonymized and analyzed qualitatively as described in the next section.

### Data Analysis

All statistical analyses were conducted using IBM SPSS Statistics for Windows (version 31.0.0). Descriptive and inferential procedures were used to summarize participant characteristics and evaluate changes across study measures. Continuous variables, including SUS scores, MPT alignment ratings, Zarit Caregiver Burden scores, and self-feeding impairment ratings, were analyzed using descriptive statistics, including means, SDs, medians, and 95% CIs to describe central tendency and variability. Categorical variables, such as demographic characteristics, professional discipline, and prior AT experience, were summarized as frequencies and percentages.

Assumptions of normality were evaluated using Shapiro-Wilk tests and visual inspection of score distributions, given the relatively small sample sizes within stakeholder groups. Shapiro-Wilk test statistics (W values) and exact *P* values were calculated and are reported for primary outcome measures. Within-subject changes from baseline to post trial were analyzed using paired-sample *t* tests, with *t* values, degrees of freedom, exact *P* values, and 95% CIs reported. For variables that did not meet assumptions of normality, or because of the ordinal nature of the self-feeding impairment ratings and small sample size, confirmatory nonparametric analyses were conducted using Wilcoxon signed-rank tests, with corresponding *Z* statistics and exact *P* values reported.

Effect sizes (Cohen *d*) were calculated to quantify the magnitude of change, using conventional benchmarks (0.2=small, 0.5=medium, and 0.8=large) [43]. Missing data were reviewed descriptively to confirm whether the missing data appeared to occur at random or if they reflected systematic withdrawal-related patterns. Given the low proportion of missing data and absence of identifiable systematic patterns, analyses were conducted using available-case data without imputation. Data were screened for potential outliers by inspecting distributions and performing range checks; no implausible values or data-entry errors requiring removal were identified.

Open-ended survey responses and interview transcripts were analyzed using thematic content analysis. Two researchers independently coded the data, iteratively refined categories, and resolved discrepancies through consensus. To support consistency in the coding language, a generative AI tool (Google Gemini, version 2.5) was used in a preliminary stage to assist with organizing the raw text and suggesting candidate theme labels. The researchers critically reviewed all AI-assisted outputs, confirmed or revised them through manual coding, and determined the final themes. Triangulation with quantitative findings and weekly audit meetings further enhanced methodological rigor [44].

### Ethical Considerations

All procedures adhered to Good Clinical Practice and ethical standards for human research. The study protocol was reviewed and approved by the WCG Clinical Institutional Review Board (IRB; Protocol #20251472). All participants, or their legal guardians in the case of minors, provided informed consent or assent prior to participation. Data collection and handling followed HIPAA-compliant procedures through the Qualtrics electronic platform. Participants who completed the follow-up survey received a US $25 electronic gift card as IRB-approved compensation for their time and effort.

## Results

### Participant Enrollment and Retention

In total, 50 individuals enrolled in the study across the stakeholder groups, reflecting a balanced distribution among providers, caregivers, and people with BUEMI. Of these, 42 (84%) participants completed posttrial surveys. Overall attrition was at 16% and was limited to posttrial survey completion rather than participation in the Obi trial itself. Participants who did not complete posttrial surveys were considered lost to follow-up after they could not be reached despite multiple contact attempts over a 2- to 3-week period following the expected survey completion date. This time frame was established to ensure that participants’ experiences with the device remained recent enough to support accurate recall.

Among providers, 13 (76%) of 17 completed all study requirements, with attrition primarily due to missed posttrial surveys. Caregivers demonstrated a comparable completion rate of 82% (14/17), with 1 early withdrawal prior to trial initiation and 2 incomplete follow-up surveys. Participants with BUEMI aged 15 years and older demonstrated full retention and 100% completion of study procedures, suggesting high engagement and feasibility of participation in this subgroup. Participants aged 5-14 years also demonstrated strong engagement, with 6/7 (86%) completing the surveys. Overall, these results indicate excellent adherence and acceptability across participant groups, particularly among patient stakeholders, with minimal losses attributable to study-related factors. Enrollment, completion, and attrition rates by stakeholder group are summarized in Table 1.

**Table 1:** Stakeholder enrollment, survey completion, and attrition.

Stakeholder groups	Participants enrolled, n	Surveys completed, n	Total dropouts and reasons for attrition
Providers	17	13	4 (1=no patient available; 3=did not complete posttrial surveys)
Caregivers	17	14	3 (1=dropped before trial; 2=did not complete posttrial surveys)
People with BUEMI^a^ (aged ≥15 years)	9	9	None—100% survey completion
Participants aged 5-14 years	7	6	1 (completed trial but no survey)
Totals	50	42	8 lost to attrition

^a^BUEMI: bilateral upper extremity motor impairment.

### Patient Demographics

Demographic and descriptive data were obtained from provider-completed Obi MDNA forms. For 1 pediatric participant whose original provider withdrew from the study, the MDNA form was instead completed by a certified ATP. In accordance with standard clinical procedures, the ATP reviewed the participant’s existing clinical documentation, including therapy notes, prior evaluations, and progress reports, and conducted a detailed video review of feeding sessions. This approach ensured that the MDNA data accurately reflected the child’s functional abilities, feeding performance, and support needs, consistent with how such assessments are typically conducted in clinical practice when direct observation by the evaluating therapist is not feasible.

Among adult and adolescent participants aged ≥15 years (n=9), the mean age was 33.9 (median 18, range 15-67) years. Gender distribution was balanced (4 males and 5 females). The most common diagnosis was cerebral palsy (n=4), followed by other neurological or neuromuscular conditions, as summarized in Table 2.

The pediatric patient cohort (n=6) ranged in age from 7 to 11 (mean 9.2, SD 1.83) years and included 4 females and 2 males. Rett syndrome was most prevalent (n=3), followed by cerebral palsy (n=2) and Charcot-Marie-Tooth disease (n=1) as listed in Table 3.

**Table 2:** Patient demographics (aged 15 years and older; n=9).

Age (years)	Birth sex	Primary diagnosis code (*ICD-10*^a^)	Diagnosis
15	Female	Q87.2	Cornelia de Lange syndrome
15	Female	G80	Cerebral palsy
48	Male	G80.9	Cerebral palsy
50	Male	G80.9	Cerebral palsy
15	Male	G80.0	Spastic quadriplegia cerebral palsy
15	Female	G11.3	Cerebellar ataxia with defective DNA repair
67	Female	G12.21	Amyotrophic lateral sclerosis
18	Male	G71.01	Duchenne muscular dystrophy
62	Female	G12.1	Spinal muscular atrophy

aICD-10: International Classification of Diseases, Tenth Revision.

**Table 3:** Patient demographics (aged <15 years; n=6).

Age (years)	Birth sex	Primary diagnosis code (*ICD-10*^a^)	Diagnosis
7	Female	F84.2	Rett syndrome
10	Male	G80.9	Cerebral palsy
9	Female	F84.2	Rett syndrome
11	Male	G80.3	Athetoid cerebral palsy
7	Female	F84.2	Rett syndrome
11	Female	G60.0	Charcot-Marie-Tooth disease

^a^*ICD-10*: *International Classification of Diseases, Tenth Revision.*

### Caregiver Characteristics

Caregiver participants (n=14) were primarily mothers (n=11), with additional representation from spouses/partners (n=2) and 1 paid caregiver. Participants were predominantly middle-aged, with varied household income levels and generally high educational attainment. Burden ratings indicated moderate to high stress, particularly related to balancing caregiving with employment and family responsibilities (9/14, 64%). Half of caregivers reported a high overall burden. Descriptive data related to the caregiver results are summarized in Table 4.

**Table 4:** Caregivers’ descriptive data (n=14).

Category and response		Value, n (%)	
Relationship to the patient			
	Mother	11 (78.6)	
	Spouse/partner	2 (14.3)	
	Paid home care staff	1 (7.1)	
Age in years			
	18-30	1 (7.1)	
	31-40	3 (21.4)	
	41-50	7 (50.0)	
	51-60	1 (7.1)	
	61-70	1 (7.1)	
	Prefer not to answer	1 (7.1)	
Annual household income (US $)			
	150,000 or more	4 (28.6)	
	100,000-149,999	3 (21.4)	
	75,000-99,999	2 (14.3)	
	50,000-74,999	0	
	25,000-49,999	1 (7.1)	
	Less than 25,000	1 (7.1)	
	Prefer not to answer	3 (21.4)	
Highest level of education attained			
	Doctoral degree	1 (7.1)	
	Master’s degree	1 (7.1)	
	Bachelor’s degree	6 (42.9)	
	Associate’s degree	4 (28.6)	
	High school graduate	1 (7.1)	
	Prefer not to answer	1 (7.1)	

### Caregiver Burden

Selected items from the Zarit Caregiver Burden Scale were used to descriptively characterize caregiver experiences and caregiving demands among participants. Caregivers reported moderate to high levels of burden across multiple domains. The greatest burden was associated with balancing caregiving responsibilities with work and family obligations (mean 2.64, SD 1.01), followed by insufficient personal time due to caregiving demands (mean 2.57, SD 1.09). Caregivers also reported that their health had been affected by caregiving responsibilities (mean 1.93, SD 1.07). Overall perceived caregiver burden averaged 2.07 (SD 1.14), with 50% (7/14) of caregivers reporting very high levels of burden. These findings suggest that participants providing feeding assistance frequently experienced substantial caregiving demands, highlighting the potential value of interventions that promote greater self-feeding independence.

### Provider Characteristics

Of 17 providers enrolled, 13 completed the study. Most were occupational therapy practitioners (n=12), and 1 was a speech-language pathologist. Providers represented outpatient clinics, private practice, and rehabilitation centers, with experience ranging from <3 to >20 years. Half of the providers were recommending Obi for the first time, and most reported limited prior integration of AT into clinical workflows. Descriptive data related to the providers who participated in this study are highlighted in Table 5.

**Table 5:** Provider demographics (n=13).

Category and response			Value, n (%)
Professional background			
	OT^a^/OTA^b^	12 (92.3)	
	SLP^c^	1 (7.7)	
Practice setting			
	Outpatient clinics	5 (38.5)	
	Private practice	3 (23.1)	
	Schools	2 (15.4)	
	Home health	1 (7.7)	
	Hospitals	1 (7.7)	
	Early intervention	1 (7.7)	
Years in professional practice			
	20 or more	4 (30.8)	
	11-20	3 (23.1)	
	7-10	3 (23.1)	
	4-6	2 (15.4)	
	2-3	1 (7.7)	
Experience recommending Obi			
	First time recommending Obi	8 (61.5)	
	Recommended 2-4 times	3 (23.1)	
	Recommended within the past year	2 (15.4)	
Education level			
	Master’s	10 (76.9)	
	Bachelors	2 (15.4)	
	Doctoral	1 (7.7)	
Experience with AT^d^			
	Rare use (<1 day/week)	7 (53.8)	
	<50% of time with AT	5 (38.5)	
	>50% of time with AT	1 (7.7)	

^a^OT: occupational therapist.

^b^OTA: occupational therapy assistant.

^c^SLP: speech-language pathologist.

^d^AT: assistive technology.

### Participants With BUEMI Self-Feeding Outcomes

**Figure 2:**
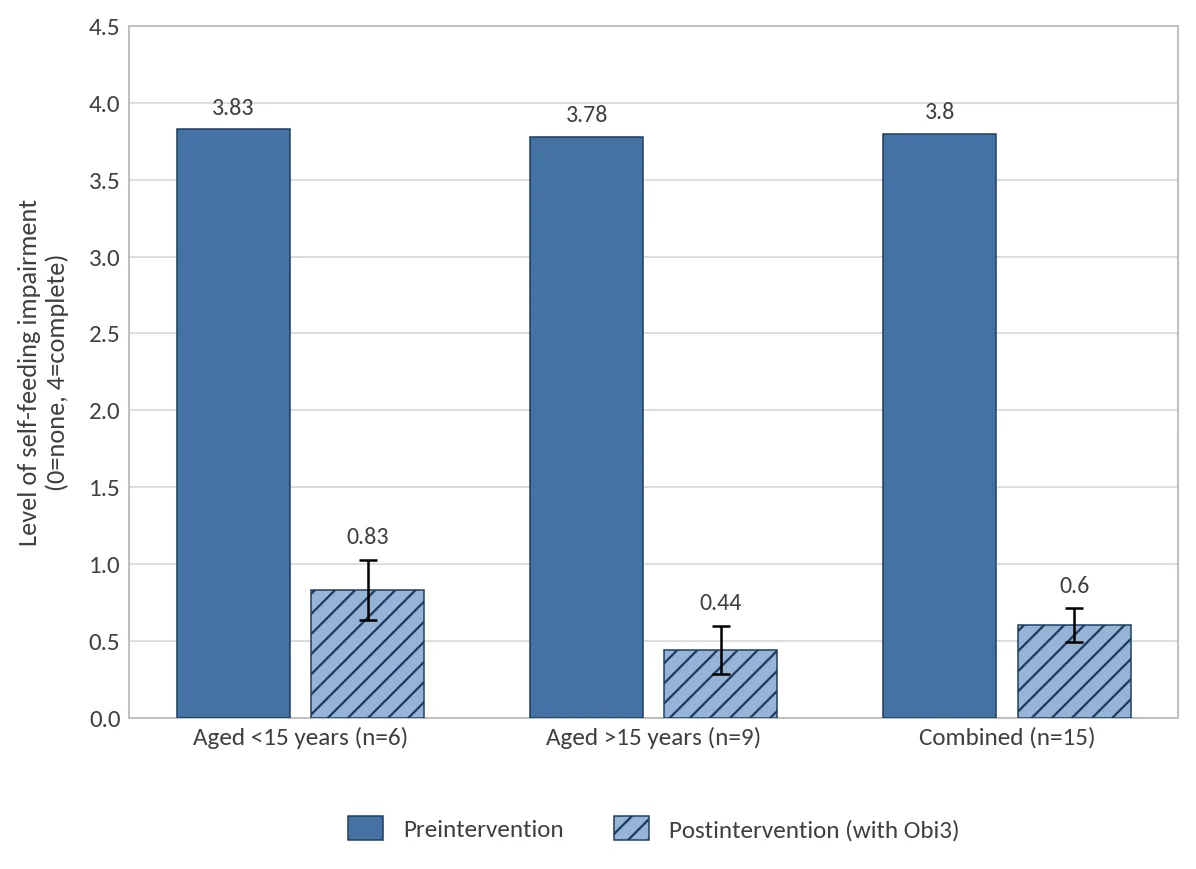
Pre- and postintervention self-feeding impairment scores using Obi3. Bar chart showing baseline and postintervention self-feeding impairment scores. Pediatric group (aged <15 years): 3.83 → 0.83; adult group (aged ≥15 years): 3.78 → 0.44; combined group: 3.80 → 0.60. All groups demonstrated large, statistically significant improvements.

Changes in self-feeding ability were measured using the Self-Feeding Impairment Scale (0=no problem to 4=complete impairment). Pediatric participants (n=6) showed significant improvement, with mean impairment decreasing from 3.83 (SD 0.41) at baseline to 0.83 (SD 0.98) post trial (*t*_5_=5.38; *P*=.002; Cohen *d*=2.37). Adult participants (n=9) demonstrated a similar pattern, improving from 3.78 (SD 0.44) to 0.44 (SD 0.53; *t*_8_=10.22; *P*<.001; Cohen *d*=4.71). Across all people with BUEMI (n=15), mean impairment decreased from 3.80 (SD 0.41) to 0.60 (SD 0.74), representing a mean reduction of 3.20 points (t_14_=10.75; *P*<.001; Cohen *d*=3.40). All participants demonstrated improvement in functional self-feeding ability following the use of Obi3 as represented in Figure 2.

### SUS Results

The SUS was administered to adult participants with BUEMI, caregivers, and providers; pediatric participants were excluded because the SUS has not been validated for children. Adult participants with BUEMI reported a mean SUS score of 82.78 (SD 17.16). Caregivers reported a mean score of 85.18 (SD 12.58), and providers reported a mean score of 85.00 (SD 10.73). Detailed results are presented in Table 6.

**Table 6:** System Usability Scale scores by stakeholder group.

Stakeholder group	n	Mean (SD)	Range	Score ≥68^a^, n (%)
Adult participants with BUEMI^b^	9	82.78 (17.16)	47.5-100	8 (88.9)
Caregivers	14	85.18 (12.58)	60-100	13 (92.9)
Providers	13	85.00 (10.73)	72.5-100	13 (100)

^a^System Usability Scale scores ≥68 are considered above the benchmark for acceptable usability. Pediatric participants did not complete the System Usability Scale.

^b^BUEMI: bilateral upper extremity motor impairment.

### Modified MPT Findings

#### Participants With BUEMI Perspectives

Survey items adapted from the MPT framework demonstrated strong alignment between Obi and user goals, competencies, and environmental supports. Adult participants (n=9) rated most items highly (*M*=4.11-4.56 on a 5-point scale) but reported lower comfort integrating Obi into daily routines (*M*=3.44) and social settings (*M*=2.50-3.78). Pediatric participants (n=6) provided consistently higher ratings (*M*=3.50-4.75), with the greatest agreement for the statements “Obi helps me achieve my feeding goals” (*M*=4.17) and “Obi improves my quality of life” (*M*=4.75). Across both groups, participants endorsed ease of use (*M*=4.50-4.56) and environmental support (*M*=4.44-4.67), indicating strong usability and goal alignment. However, additional research is warranted to examine how Obi can further facilitate integration into daily routines and community participation.

#### Caregiver Outcomes

Caregivers reported reduced burden and stress, with increased time for personal responsibilities and less physical fatigue during meals. Open-ended feedback emphasized Obi’s dual benefit, enhancing user independence while easing caregiver workload. Common suggestions included improving portability for travel, stabilizing switch ports, adding visual or auditory cues for users with sensory impairments, and optimizing utensil scooping performance. While “reward mode” was praised by some for motivation, others found it confusing, suggesting clearer instructions or customization options. Overall sentiment was highly positive, with several caregivers describing Obi as “life-changing” for both users and families.

#### Provider Perspectives

Providers described Obi3 as highly beneficial, easy to use, and clinically valuable. They highlighted smooth operation, intuitive setup, and clear instructional materials, particularly the Quick Start Guide and online tutorials. Suggested refinements included utensil geometry, bite consistency, and environmental adaptability. Several providers emphasized that Obi3 promoted measurable independence and engagement among participants with BUEMI, with one describing it as “a necessary tool for promoting autonomy.”

### Safety Findings

No adverse events, device deficiencies, or unanticipated problems were reported during the study. The absence of safety incidents supports Obi’s favorable safety profile, consistent with its FDA Class I designation and regulatory history.

### Follow-Up Qualitative Interviews

#### Overview

To complement the survey data and gain a deeper understanding of usability experiences, follow-up virtual interviews were conducted. Qualitative data from these semistructured interviews were analyzed to enrich, validate, and extend the quantitative findings. Interview participants included caregivers, health care providers, and clients who had previously used the Obi3 feeding device and completed the corresponding surveys. A total of 7 participants (3 participants with BUEMI, 1 caregiver, and 3 providers) completed follow-up virtual interviews to expand on survey findings.

Participants with BUEMIs ranged in age from 11 to 48 years, with primary diagnoses of spinal cord injury (quadriplegia), cerebral palsy, and Charcot-Marie-Tooth disease. Two participants were adults (aged 35 and 48 years), and 1 was a minor (aged 11 years) who participated with support from her mother; all clients completed follow-up interviews. The caregiver participant was a mother aged 31-40 years with a doctoral degree and an annual household income exceeding US $150,000; she also completed a follow-up interview. Providers included an occupational therapist and a speech-language pathologist, both with master’s degrees. Their years of practice ranged from 2-3 years to more than 20 years, and their current settings included an outpatient clinic, private practice, and a state developmental disabilities resource center. Overall, 2 providers worked in Louisiana and 1 in Nebraska; 2 had prior experience recommending Obi. Time dedicated to AT varied, with one provider spending less than 50% but more than 20% of their time on AT, another about 20%, and the speech-language pathologist 50%-75%.

The following themes emerged from this analysis:

#### Theme 1: Enhanced Independence and Quality of Life

This theme highlights the significant positive impact of Obi3 on the lives of users and their families. Clients, including a client with quadriplegia and a young child, reported a newfound sense of autonomy and freedom during mealtimes. One adult client appreciated being able to eat on their own terms and time, rather than relying on a caregiver for every bite, which provided a mental and emotional boost. A provider noted that their client became tearful at the thought of the device being taken away, indicating a profound improvement in their quality of life. Furthermore, the device facilitated social interaction during meals, as it allowed a client and their caregiver to sit and talk together while eating, transforming mealtimes from a task into an enjoyable, social experience.

The timing was perfect for the feeding because I was capable of pressing the button and giving the bite when I wanted it.

adult patient

#### Theme 2: Usability and Ease of Setup

Participants generally found the Obi3 to be user-friendly and easy to set up. A caregiver described the initial setup as “not frustrating” and straightforward after calibrating it with the manual. A provider, who is a therapist, reported that they learned to use the device in under 30 minutes and found it “super easy” and “very user-friendly,” even for caregivers who may not have extensive technical knowledge. The *Quick Start Guide* and online resources were also praised for being helpful. However, some participants, including a provider, experienced challenges with certain button functionalities, such as getting the device to start or enter training mode, suggesting a need for clearer instructions.

It was very, very user-friendly. I was able to show three of his caregivers...And it took us less than 10 minutes to get it set up.

provider

#### Theme 3: Portability and Design Limitations

Both caregivers and clients identified the device’s size and lack of portability as a major limitation. A caregiver expressed a need for a smaller, more portable version for use outside the home, such as at restaurants, church dinners, or school. An adult client shared a similar sentiment, stating they would not feel comfortable taking the large device into a public restaurant. The client likened bringing the device to a “briefcase” that takes up half the table, which they felt detracted from the social experience. A provider also mentioned challenges in finding a suitable table to accommodate the device in a clinical setting.

Obi was a little too big for the wheelchair tray. Like, we made it work, but um if it were a little smaller, I think it would have worked better.

caregiver

#### Theme 4: Functional Improvements and Feature Suggestions

Participants offered specific suggestions to improve the device’s functionality and versatility. The need for additional utensils, such as a fork, a spoon, and a dull knife for cutting food, was raised by a caregiver. Another client and a caregiver noted the issue of the spoon frequently coming up empty, suggesting that the device should be programmed to always use the bowl’s edge to ensure a successful scoop. Different plate sizes, including a larger bowl option, were also recommended. A provider highlighted the benefit of the smaller spoon in the Obi3 version for a client with a small mouth and choking concerns. The device’s ability to handle liquids was also a significant benefit for clients with dehydration issues.

The machine was easy to use, but the spoon couldn’t scoop food from the edge, so I think it needs a larger circumference.

adult patient

#### Theme 5: Motivating Impact and Self-Advocacy

The device was found to be highly motivating for both clients and their families. A pediatric patient loved using the device and was excited to show it to others, even wanting to make a YouTube video about it. A provider noted that the device was motivating for both a child user and their parent, helping them feel that success with the device was achievable at home. One client even used the device to express their food preferences by immediately dumping food they did not want and giving a different choice. This demonstrates that the device empowers users to exercise greater control and self-advocacy over their mealtimes.

The Obi device helped reduce my caregiving workload during mealtimes by allowing my child...to self-feed. The Obi device has the potential to be a ‘game changer’ for our family, as we have been hand-feeding our child three meals a day for ten years.

caregiver

## Discussion

### Principal Findings

Obi3 demonstrated high usability and short-term functional improvements in self-feeding performance under real-world conditions. Statistically and clinically significant gains were observed across all patient participants, who progressed from severe or complete self-feeding impairment at baseline to mild or no impairment after a 1-week home trial. These improvements indicate that Obi3 successfully fulfills its intended medical function, compensating for the loss of voluntary upper-limb movement and enabling users to independently select and consume food and liquid. These findings support the feasibility of the device as an assistive intervention; however, longer-term and controlled studies are needed to establish sustained clinical efficacy, nutritional outcomes, and psychosocial impact.

Quantitative outcomes were reinforced by qualitative and observational data. SUS scores exceeded the industry benchmark of 68 across participants with BUEMI (mean 82.8, SD 17.2), caregivers (mean 85.2, SD 12.6), and providers (mean, SD 10.7), demonstrating a consistent pattern of high perceived usability, reliability, and ease of clinical integration. Complementary survey findings aligned with the MPT framework revealed strong alignment among device capabilities, user goals, and environmental supports, including key predictors of sustained adoption in real-world contexts. Triangulation with interview data further confirmed that participants experienced enhanced autonomy, dignity, and reduced caregiver burden. Collectively, these findings demonstrate that Obi3 meets essential design and performance criteria for DME, addressing not only the physical restoration of self-feeding but also the psychosocial and participatory dimensions of independence.

Participant feedback regarding utensil options and food handling underscores the importance of caregiver and provider education on optimal food presentation when using robotic feeding devices. Although a caregiver suggested adding utensils such as forks or dull knives to facilitate food cutting, this feedback reflects a potential misunderstanding of the device’s intended use. As with most adaptive feeding interventions, foods presented for robotic delivery should already be prepared in appropriately sized, manageable portions consistent with each user’s swallowing safety and motor abilities. Proper meal preparation, including cutting food into uniform, bite-sized pieces and selecting suitable textures, ensures safe, reliable spoon loading and reduces the risk of choking or aspiration.

The *Instructions for Use* provided with Obi3 include guidance on food size, placement, and consistency to promote optimal performance; however, these directions may not always be sufficiently emphasized or understood by all users. Some caregivers may benefit from more explicit visual examples or in-person demonstrations to reinforce these principles and ensure consistent adherence to safe feeding practices. Integrating this training into initial device setup, clinician-guided instruction, and follow-up sessions could enhance device performance, minimize user frustration, and strengthen caregiver confidence. Ultimately, reinforcing food-preparation education is essential to achieving safe, effective, and independent mealtime participation with robotic feeding systems.

### Comparison With Prior Work

Unlike other commercially available powered feeding systems, Obi3 uses a 6-DOF robotic arm that produces naturalistic utensil trajectories and customizable positioning, allowing accommodation for diverse user anatomies and dining environments. These technical advancements correspond with prior evidence from Al-Halimi and Moussa [28], who demonstrated that users with severe motor impairments could complete complex activities of daily living, including self-feeding, using a 6-DOF robotic arm. This study also builds upon earlier work by Burgos [33], which reported improved independence among 19 Obi users in home and clinical contexts, by expanding the sample to a larger, more diverse group and incorporating caregiver and provider perspectives.

The observed reductions in caregiver workload and stress mirror findings from studies of adaptive eating devices and robotic assistance in neurological populations [12,13], underscoring the dual benefit of such technologies for users and care partners alike. In addition, the absence of adverse events aligns with existing safety records in the FDA’s Manufacturer and User Facility Device Experience [31] and the European Database on Medical Devices [32], supporting Obi3’s favorable benefit-to-risk profile. Consistent with principles of human-centered design [36], the findings affirm that usability, environmental fit, and psychosocial relevance are critical determinants of long-term adoption and meaningful engagement with assistive technologies.

### Limitations

Several limitations should be considered when interpreting these findings. First, data collection primarily relied on self-report surveys, which are well-suited for capturing perceptions of usability, satisfaction, and caregiver burden, but are inherently subject to recall bias, social desirability bias, and individual differences in self-awareness or communication ability. Follow-up interviews were incorporated to contextualize these responses and reduce misinterpretation.

Although 50 participants were enrolled and 42 completed all study requirements, the sample size remains relatively small, limiting the ability to detect subgroup differences or perform multivariate analyses. Although the target enrollment of 60 participants was not achieved, the final sample of 50 participants exceeded typical sample sizes reported in usability studies and was considered sufficient to address the study objectives. While the number of participants aligns with the FDA’s guidance for formative and summative usability testing, larger multisite studies would enhance the external validity and representativeness of findings. Future research should include greater demographic, diagnostic, and cultural diversity, as well as participants using alternative switch interfaces or mounting configurations to evaluate device performance across varied clinical and environmental contexts.

The self-feeding impairment rating scale used in this study was a study-specific clinical measure embedded within the MDNA assessment process and has not undergone formal psychometric validation or interrater reliability testing. Although structured scoring anchors, clinician-guided assessment procedures, and repeated ratings by the same provider were used whenever feasible to support consistency, the measure was intended primarily to descriptively characterize functional change during the Obi3 usability trial rather than to serve as a definitive clinical outcome instrument. Consequently, the observed improvements in self-feeding performance should be interpreted as preliminary evidence of functional feasibility rather than conclusive evidence of clinical efficacy. In contrast, the SUS, which served as the primary standardized usability outcome measure, is a well-established, psychometrically validated instrument widely used in health and AT research.

The study was conducted over a short-term trial period (1 week), which may not fully capture the effects of sustained device use on long-term outcomes such as user satisfaction, skill acquisition, changes in muscular strength or tone, or caregiver burden. Extended longitudinal and home-based evaluations are warranted to understand the potential impact on health outcomes, maintenance needs, and adherence over time.

The device manufacturer sponsored this study, and several authors maintain professional or financial relationships with the sponsor. Although these relationships were fully disclosed and reviewed by the IRB, they introduce a potential risk of bias in study design, data interpretation, and reporting. To mitigate this risk, data collection followed standardized protocols. Ratings of participants’ self-feeding performance were completed by licensed therapists, who were also study participants, using a structured clinical data collection instrument (Obi MDNA) consistent with routine practice. All survey data were collected remotely via the Qualtrics platform, with participants entering responses independently and without direct interaction with the researchers responsible for data analysis. Qualitative interviews were conducted by a researcher independent of the sponsor, and data analysis incorporated dual independent coding, triangulation, and audit procedures. Additionally, all authors had access to aggregated study data, and statistical analyses were independently verified. Finally, this investigation did not include objective nutritional or biomechanical measures (eg, meal duration, food intake volume, posture, or reach analysis), which could strengthen the interpretation of functional and clinical outcomes. Integrating such measures in future studies would allow for a more comprehensive understanding of the device’s impact on independence, health, and participation across stakeholder groups.

## Abbreviations

AT: assistive technology
ATP: assistive technology professional
BUEMI: bilateral upper extremity motor impairment
DME: durable medical equipment
DOF: degree of freedom
FDA: US Food and Drug Administration
HIPAA: Health Insurance Portability and Accountability Act
ICF: International Classification of Functioning, Disability, and Health
IRB: institutional review board
ISO: International Organization for Standardization
MDNA: Medical Device Needs Assessment
MPT: Matching Person and Technology
SUS: System Usability Scale
